# Brassinosteroid biosynthesis gene *OsD2* is associated with low-temperature germinability in rice

**DOI:** 10.3389/fpls.2022.985559

**Published:** 2022-09-20

**Authors:** Sun Ha Kim, Kyu-Chan Shim, Hyun-Sook Lee, Yun-A Jeon, Cheryl Adeva, Ngoc Ha Luong, Sang-Nag Ahn

**Affiliations:** ^1^Department of Agronomy, College of Agriculture and Life Sciences, Chungnam National University, Daejeon, South Korea; ^2^Crop Breeding Division, National Institute of Crop Science, Wanju-Gun, South Korea

**Keywords:** rice, *Oryza sativa* L., *Oryza rufipogon*, low-temperature germination, *OsD2*, brassinosteroids, cytochrome P450

## Abstract

In rice, low-temperature germinability (LTG) is essential for stable stand establishment using the direct seeding method in temperate and high-altitude areas. Previously, we reported that the quantitative trait locus *qLTG1* is associated with LTG. *qLTG1* is also associated with tolerance to several abiotic stresses, such as salt and osmotic conditions. In this study, map-based cloning and sequence analysis indicated that *qLTG1* is allelic to *DWARF2* (*OsD2*), which encodes cytochrome P450 D2 (LOC_Os01g10040) involved in brassinosteroid (BR) biosynthesis. Sequence comparison of the two parental lines, Hwaseong and *Oryza rufipogon* (IRGC 105491), revealed five single nucleotide polymorphisms (SNPs) in the coding region. Three of these SNPs led to missense mutations in *OsD2*, whereas the other two SNPs were synonymous. We evaluated two T-DNA insertion mutants, *viz.*, overexpression (*OsD2*-OE) and knockdown (*OsD2*-KD) mutants of *OsD2*, with the Dongjin genetic background. *OsD2*-KD plants showed a decrease in LTG and grain size. In contrast, *OsD2*-OE plants showed an increase in grain size and LTG. We also examined the expression levels of several BR signaling and biosynthetic genes using the T-DNA insertion mutants. Gene expression analysis and BR application experiments demonstrated that BR enhanced the seed germination rate under low-temperature conditions. These results suggest that *OsD2* is associated with the regulation of LTG and improving grain size. Thus, *OsD2* may be a suitable target for rice breeding programs to improve rice yield and LTG.

## Introduction

Rice is one of the most important cereal crops in the world, and its production needs to be increased to meet the demands of an increasing population. However, resource limitations, such as decreased agricultural land, water supply, and poor soils, are major challenges in many rice producing countries (Kumar and Ladha, 2011).

Strong seedling vigor under low-temperature conditions is an important objective of rice breeding programs using direct seeding cultivation methods. The direct seeding method is widely used because it requires less labor, water, and energy than transplanted cultivation systems (Hyun et al., 2015). However, low-temperature stress retards seed germination in direct seeding cultivation, especially in temperate regions, tropical and subtropical areas at high altitudes, and areas that use cold irrigation water (Fujino et al., 2008). Germinability and early seedling growth are major components of seedling vigor, and improving germinability under low-temperature conditions can lead to improved seedling vigor (Iwata et al., 2010). A wide range of phenotypic variations of low-temperature germinability (LTG) has been observed in rice accessions (Wang et al., 2018).

It has been demonstrated that LTG is controlled by multiple genes, and over 30 LTG quantitative trait loci (QTL) have been identified using biparental mapping populations (Miura et al., 2001; Fujino et al., 2004; Chen et al., 2006; Li et al., 2013b; Jiang et al., 2017). Among these QTLs, the major QTL, *qLTG3-1*, was cloned using a population derived from a cross between Italica Livorno and Hayamasari (Fujino et al., 2008). The expression pattern of *qLTG3-1* suggested that the qLTG3-1 protein may function to weaken the tissues covering the embryo during germination. *OsSAP16* (*stress-associated protein 16*), which encodes a zinc finger domain protein, was cloned from a genome-wide association study (GWAS; Wang et al., 2018). Loss of function of *OsSAP16* led to decreased LTG levels, and the expression level of *OsSAP16* was correlated with LTG in the rice core collection. Although many other QTLs for LTG have been identified, knowledge of the molecular functions of QTL and gene characterization is still poor.

*OsD2* (LOC_Os01g10040) has been previously cloned as *ebisu dwarf 2* (*d2*) gene (Hong et al., 2003; Sakamoto et al., 2012; Li et al., 2013a; Fang et al., 2016). This gene encodes cytochrome P450 and functions in the brassinosteroid (BR) biosynthesis pathway. Two mutants (*d2-1* and *d2-2*) with single nucleotide polymorphism (SNP) in the *OsD2* exon region have been characterized, and these two *d2* mutants showed reduced plant height with erect leaves (Hong et al., 2003). Many *OsD2* mutants have been reported, but most of the mutant alleles cause unfavorable phenotypes, such as severe dwarf and small grain size, leading to a decrease in yield. The *OsD2* mutants *chromosome segment deleted dwarf 1* (*csdd1*) and *small grain 11* (*smg11*) showed severe dwarf phenotype and small grain with mild dwarf phenotype, respectively, in a *japonica* background (Li et al., 2013a; Fang et al., 2016). An investigation of two independent T-DNA insertion mutants of *OsD2* showed that T-DNA homozygous insertion led to the loss of function of *OsD2*, resulting in a severe dwarf phenotype (Li et al., 2013a).

Brassinosteroids are plant hormones that have pleiotropic effects; they regulate seed germination, plant growth and development, rhizogenesis, senescence, and leaf abscission (Anuradha and Rao, 2001). The positive effects of BR under stress conditions have been reported in monocot and dicot plant species. BR application ameliorated osmotic stress in germinating seeds and promoted seedling growth in three sorghum varieties (Vardhini and Rao, 2003). Similarly, exogenous BR treatment improved the germination rate of cucumber seeds under salinity stress (Wang et al., 2011). Treatment with 24-epibrassinolide and 28-homobrassinolide, increased the germinability of *indica* rice IR64 under saline conditions (150 mM NaCl; Anuradha and Rao, 2001). Although stress tolerance at the germination stage has been reported in many plant species, studies on the effect of BR on LTG are limited.

Previously, we identified five QTLs for LTG using a population derived from the interspecific cross between the Korean *japonica* cultivar “Hwaseong” and *Oryza rufipogon* (IRGC 105491; Nguyen et al., 2012). *Oryza rufipogon* alleles of the five QTLs increased germination rate under low-temperature conditions. Among these QTLs, *O. rufipogon* alleles at *qLTG1* and *qLTG3* additively increased LTG (Shim et al., 2020). *qLTG1* was narrowed to a 167-kb region with 18 candidate genes for LTG (Shim et al., 2019). In the present study, we identified *OsD2* (LOC_Os01g10040) as a candidate gene for *qLTG1*. Two T-DNA insertion mutants were used to confirm the association between *OsD2* and LTG expression. *OsD2* encodes a cytochrome P450 protein involved in BR biosynthesis. To determine the relationship between BR and LTG, BR-feeding experiments were conducted at the germination stage under low-temperature conditions. The expression levels of BR signaling and biosynthesis genes were examined to understand the molecular mechanisms of the BR pathway. Gene expression analysis and BR application experiments demonstrated that BR enhanced the seed germination rate under low-temperature conditions. These data suggest that *OsD2* is associated with the regulation of LTG and that the *O. rufipogon OsD2* allele is a suitable option for improving LTG in rice.

## Materials and methods

### Plant materials and growth condition

In a previous study, 96 introgression lines (ILs; TR1–96) were developed from an interspecific cross between the Korean *japonica* line Hwaseong and *O. rufipogon* (Yun et al., 2016). QTL analysis for LTG using these 96 ILs revealed five QTLs, including *qLTG1* (Nguyen et al., 2012). To fine-map *qLTG1*, two introgression lines (TR5 and TR20) harboring the *O. rufipogon qLTG1* allele in the Hwaseong background were crossed with Hwaseong, and 971 F_2_ plants were developed. The F_2:3_ line was used for substitution mapping of *qLTG1*. Two T-DNA insertion lines (PFG-3A-07238 and PFG-3A-07287) in *OsD2* genic region were obtained from Kyung Hee University, Yongin, South Korea (Jeon et al., 2000; Jeong et al., 2006), and the T-DNA insertion was confirmed using two sets of PCR primers (Supplementary Table 1). From these T-DNA lines, we selected plants heterozygous at the T-DNA locus because plants homozygous for the T-DNA insertion showed a severe dwarf phenotype with no seed setting (Supplementary Figure 1). We tested the genotypes of 40 seeds from heterozygous plants and observed 1:2:1 ratio of no T-DNA insertion, heterozygous and homozygous insertion, respectively suggesting that there is no segregation distortion at this locus (χ^2^ = 0.105, 0.95 ≥ *p* ≥ 0.90). To investigate the sequence variations in *OsD2*, 96 rice accessions from the KRICE_CORE set were examined (Kim et al., 2016; Supplementary Table 2). Plant materials for fine-mapping and T-DNA insertion lines were sown in mid-April, and 30-day-old seedlings were transplanted into a paddy field at Chungnam National University, Daejeon, South Korea. Furthermore, 96 rice accessions from the KRICE_CORE set were grown in an experimental field at Chungcheongnam-do Agricultural Research and Extension Services (CNARES), Yesan, South Korea. Data of agronomic traits of Hwaseong, TR5, and TR20 were used from our previous study (Yun et al., 2016).

### DNA extraction and genotype analysis

DNA was extracted from fresh leaf tissues using the method described by Causse et al. (1994). PCR was carried out as described by Jeon et al. (2018), with minor modifications: 95°C for 5 min, followed by 35 cycles at 95°C for 30 s, 55–58°C for 30 s, and 72°C for 30 s, and 5 min at 72°C for the final extension. PCR products were separated on a 2–3% agarose gel stained with StaySafe Nucleic Acid Gel Stain (RBC, New Taipei City, Taiwan). For *qLTG1* fine-mapping, InDel markers were designed (Supplementary Table 1). T-DNA insertion genotyping was conducted using two sets of primers (Supplementary Table 1).

### Evaluation of low-temperature germination

Germination tests were conducted as described by Nguyen et al. (2012) and Shim et al. (2019, 2020), with minor modifications. Seeds were harvested 45 days after flowering, and dried in a greenhouse for 2 weeks. Seeds from two T-DNA insertional heterozygous lines were harvested and used for germination test. To break dormancy, seeds were incubated at 55°C for 3 days. To confirm the breakage of seed dormancy, 20 seeds were germinated in duplicates at optimal germination temperature (28°C) and dark conditions. For the LTG test, 20 seeds were placed in a 60 mm petri dish with filter paper and filled with 5 ml of distilled water. Seeds were incubated in triplicates at 13°C and dark conditions. Seeds were considered germinated when the seed coat was broken and the white embryo emerged. Germinated seeds were counted, and the germination percentage (%) was calculated. All germination experiments were conducted three times.

### *OsD2* haplotype analysis

Haplotype analysis was carried out using KRICE_CORE set genotype data from the Kongju National University, Yesan, South Korea (Kim et al., 2016, Supplementary Table 2). LTG phenotype data were obtained from our previous study (Shim et al., 2020).

### Feeding experiment

To examine germination under stress conditions, seed germination tests were conducted using Hwaseong, *O. rufipogon,* and TR5 under salinity (250 mM NaCl) and high-osmotic (500 mM mannitol) conditions. Seeds were incubated at 28°C for 7 days under each stress condition.

To determine whether BR is associated with LTG, low-temperature germination tests were carried out under 1 μM 24-epi-brassinolide (eBL; Sigma-Aldrich Co., MO, United States) and 1 μM brassinazole (BRZ; Sigma-Aldrich Co., MO, United States) treatment conditions. T-DNA insertion lines (*OsD2*-KD and *OsD2*-OE) were used for the BR treatment test, and 25 seeds were incubated in triplicates at 13°C for 7 days. Germinated seeds were counted, and the germination rate (%) was calculated daily after incubation. All germination experiments were repeated three times.

### RNA isolation and real time quantitative reverse transcription PCR

Total RNA was isolated from seeds at the germination stage or from 7-day-old seedlings using a NucleoSpin RNA kit (Macherey Nagel, Deuren, Germany), according to the manufacturer’s instructions. Following reverse-transcription into the first-strand cDNA using SmartGene Mixed cDNA synthesis kit (SJ Bioscience, Daejeon, Korea), real-time PCR was performed using a CFX Connect Real-Time System (Bio-Rad, CA, United States). The real-time PCR protocol and conditions were as described by Jeon et al. (2021), with minor modifications: 15 min at 95°C to denature and activate the enzyme, followed by 40 cycles at 95°C for 20 s, 60–55°C for 40 s (depending on primer annealing temperature), and 72°C for 30 s. The quantitative reverse transcription PCR (qRT-PCR) data were analyzed using the 2^–∆∆Ct^ method. *Rice tumor-expressed protein* (*OsTMP*) was used for normalization, and relative expression levels were compared using Student’s *t*-test. The primers used in this study are listed in Supplementary Table 1.

### Statistical analysis

One-way ANOVA and Tukey’s test were carried out using Minitab19 software. Student’s *t*-test was performed using Microsoft Excel software.

## Results

### Comparison of germinability under stress conditions

**Figure 1:**
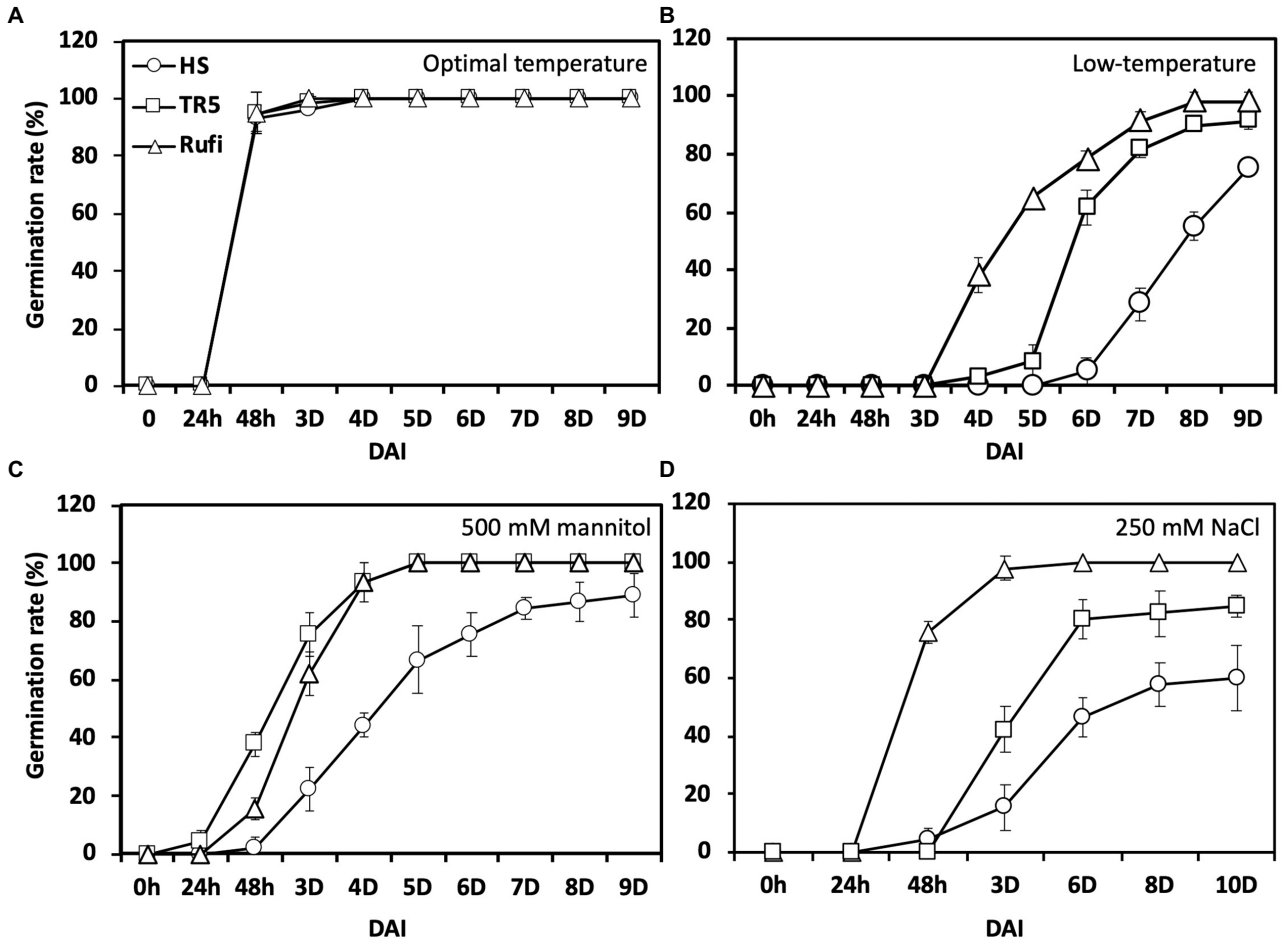
Seed germination under low-temperature and abiotic stress conditions using Hwaseong, *Oryza rufipogon*, and TR5. **(A)** Germination rate under optimal temperature (28°C); **(B)** Germination rate under low-temperature (13°C); **(C)** Germination rate under high osmotic stress (500 mM mannitol) at 28°C; and **(D)** Germination rate under high salinity stress (250 mM NaCl) at 28°C. Triangle, square, and circle indicate *O. rufipogon*, TR5, and Hwaseong, respectively. Data are presented as mean ± SD (*n* = 3). DAI, days after incubation.

Three accessions, Hwaseong, *O. rufipogon*, and the introgression line TR5 harboring *qLTG1*, were tested for germinability under three stress conditions. Initially, the germination rate was evaluated at 28°C to check seed dormancy. All lines showed a 100% germination rate 4 days after incubation (DAI), indicating that seed dormancy was broken (Figure 1A). Then, we compared LTG at 13°C, and *O. rufipogon* showed the highest LTG, followed by TR5 and Hwaseong (Figure 1B). Subsequently, germinability was evaluated under osmotic and salinity stress conditions at 28°C. In the 500 mM mannitol treatment, *O. rufipogon* and TR5 plants showed similar germination patterns, and their germination rates were higher than that of Hwaseong plants (Figure 1C). The highest germination rate was observed in *O. rufipogon* under the 250 mM NaCl treatment (Figure 1D). The germinability of TR5 was higher than that of Hwaseong but lower than that of *O. rufipogon*. These results indicate that the *O. rufipogon qLTG1* allele increased germinability at low temperatures, as well as under osmotic and salinity stress conditions.

### Fine-mapping of *qLTG1*

**Figure 2:**
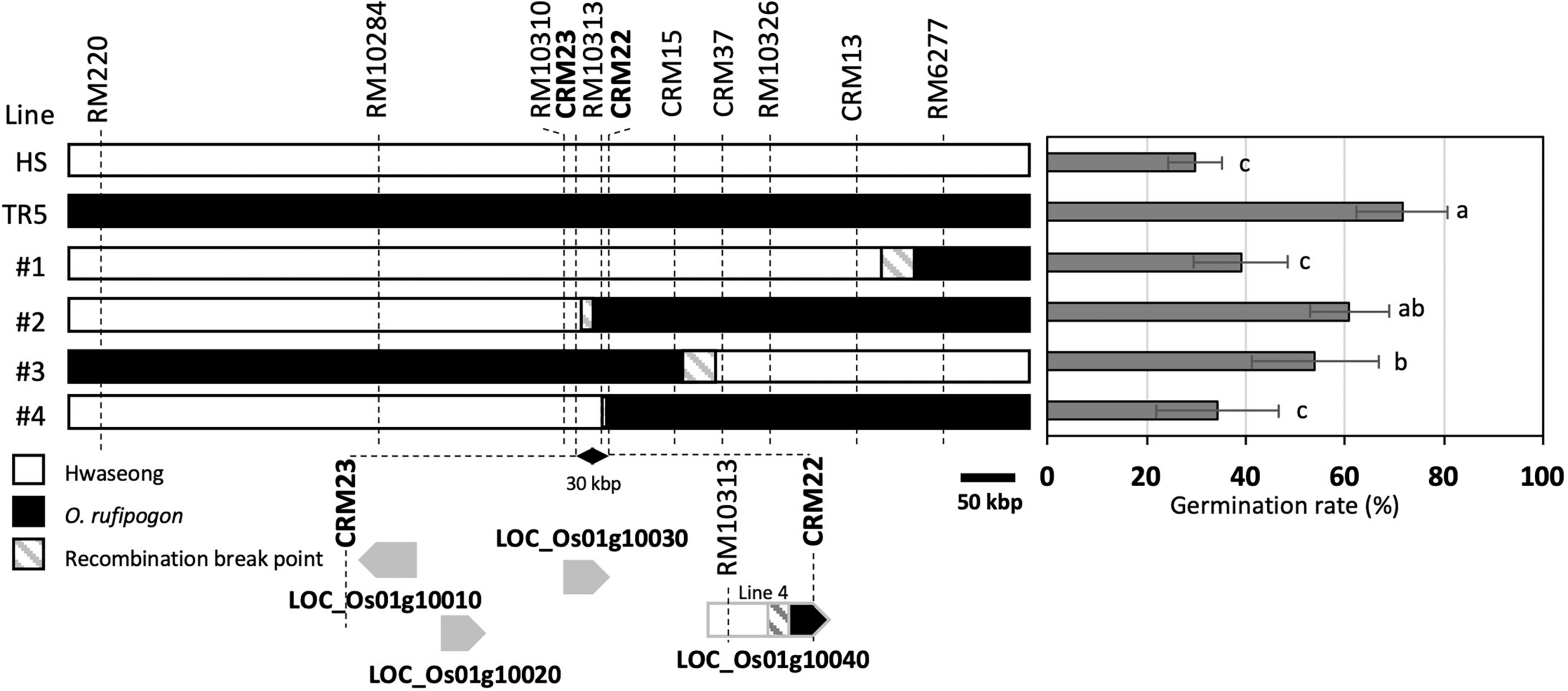
Substitution mapping of *qLTG1*. Germination percentage was measured at 7 days after incubation. Error bars indicate the SD of the mean. Different letters to the right of the bar graph indicate significant differences at *p* < 0.05, based on Tukey’s test.

To narrow down the *qLTG1* locus, we fine-mapped the *qLTG1* region using recombinant plants. Four recombinants with breakpoints between two markers, RM220 and RM6277, were observed in 971 F_2_ plants; these plants were then self-pollinated to produce F_3_ homozygous lines (Figure 2). To determine the exact breakpoint in the four recombinants, newly designed InDel markers (CRM1–40) were used. The line 4 was informative in determining the target region of the candidate genes because the LOC_Os01g10040 allele of line 4 was a hybrid between those of Hwaseong and *O. rufipogon.* This line showed significantly lower germination rate than TR5. Sequencing LOC_Os01g10040 of the line 4 revealed that the chromosome recombination point was between the fourth intron and the seventh intron suggesting that LOC_Os01g10040 of the line 4 allele was not as effective as *O. rufipogon* in LTG and this gene should be included as candidate gene for *qLTG1*. Finally, *qLTG1* was delimited to a 30-kb region between CRM23 and CRM22. Based on the Nipponbare reference genome, this region contains four genes (Figure 2; Table 1).

**Table 1:** List of candidate genes in the *qLTG1* region.

Gene ID	Description
LOC_Os01g10010	Prenylated rab acceptor
LOC_Os01g10020	Ectonucleotide pyrophosphatase/phosphodiesterase family member 1
LOC_Os01g10030	Hypothetical protein
LOC_Os01g10040	Cytochrome P450, Brassinosteroids biosynthesis, Regulation of plant architecture

### Candidate gene analysis

The fine-mapping results indicated the existence of four genes (LOC_Os01g10010, LOC_Os01g10020, LOC_Os01g10030, and LOC_Os01g10040) at the *qLTG1* locus. Based on release 7 of the MSU Rice Genome Annotation Project on rice IRGSP-1.0 genome,^1^ annotations of these four genes were as follows: the prenylated Rab acceptor for LOC_Os01g10010, ectonucleotide pyrophosphatase/phosphodiesterase family member 1 for LOC_Os01g10020, hypothetical protein for LOC_Os01g10030, and cytochrome P450, BR biosynthesis, and regulation of plant architecture for LOC_Os01g10040 (Table 1). Four candidate genes have sequence variation between Hwaseong and *O. rufipogon* including synonymous and non-synonymous variations (Supplementary Tables 3, 4).

To select the candidate gene for *qLTG1*, gene expression levels were examined at four different time points during seed germination (0, 24, 48, and 72 h after incubation) at 13°C in Hwaseong, *O. rufipogon*, and TR5 (Supplementary Figure 2). Among the four genes, LOC_Os01g10040 displayed a consistent expression pattern during germination in the three lines. The expression of LOC_Os01g10040 in *O. rufipogon* and TR5 was higher than that in Hwaseong 24 and 48 h after incubation, whereas no significant difference was observed at 72 h. Compared with LOC_Os01g10040, the expression patterns of the other three genes were inconsistent. These results strongly suggest an association between LOC_Os01g10040 and LTG. Several studies have reported that LOC_Os01g10040 (*OsD2*) regulates several key agronomic traits, including plant height, leaf angle, and grain size (Hong et al., 2003; Li et al., 2013a; Fang et al., 2016). Based on these findings, *OsD2* was chosen as the candidate gene for further analysis.

### Sequence comparison of *OsD2* between Hwaseong and *Oryza rufipogon*

The sequence of *OsD2* was compared between Hwaseong and *O. rufipogon* (Supplementary Figure 3; Supplementary Table 4). The size of the *OsD2* genomic sequence and coding sequence were 7,783 and 1,473 bp, respectively, and several sequence differences were observed between Hwaseong and *O. rufipogon*. Although many differences were detected in the genomic sequences, we focused only on the exon region. Five SNPs were found in the exon region; the SNPs were located on the first, fourth, and seventh exons. Among the five SNPs, three were non-synonymous; these SNPs (+76_A/G, +1706_C/T, and + 1726_G/A) changed amino acids from Gly, Leu, and Met (Hwaseong) to Ser, Pro, and Val (*O. rufipogon*), respectively (Supplementary Figure 3A). The first non-synonymous SNP (+76_A/G) was located on the membrane anchor domain, whereas the other two were not located on known protein domains (Supplementary Figure 3B). Additionally, 14 sequence variants were observed in the promoter region (Supplementary Table 4).

### *OsD2* controls LTG

**Figure 3:**
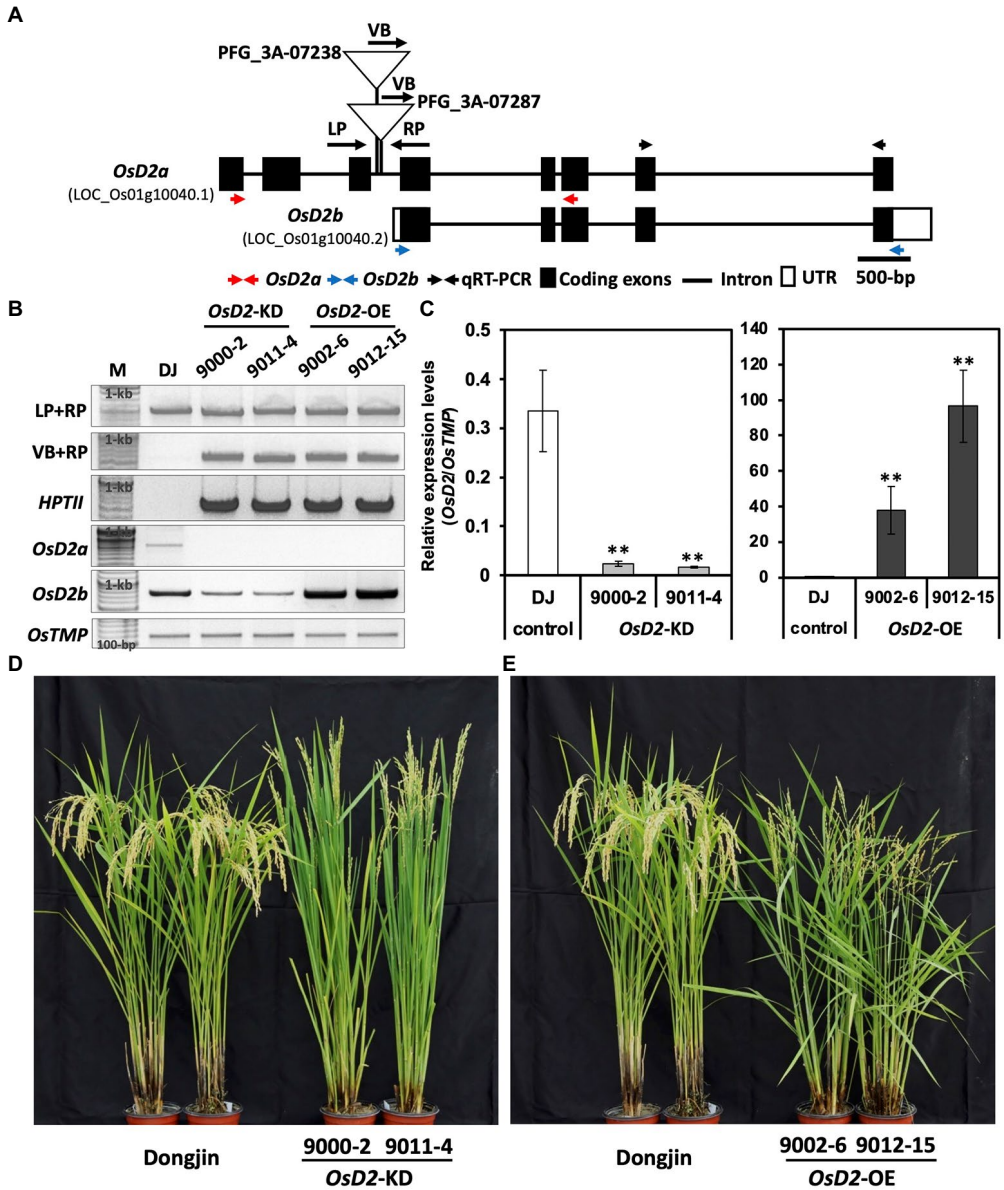
Characterization of T-DNA insertion lines. **(A)** Gene structure of *OsD2* and T-DNA insertion sites in two lines. VB, vector border; LP, left primer; and RP, right primer. **(B)** PCR amplicons to confirm T-DNA insertion together with hygromycin resistant gene (*HPTII*) and PCR amplicons for transcript form confirmation of *OsD2a* and *OsD2b* using cDNA. M: 100-bp size marker, DJ: Dongjin. **(C)** Gene expression level of *OsD2* in the T-DNA insertion lines. *OsTMP* was used for gene normalization. Error bars indicated SD. ** indicates a significant difference between each T-DNA insertion line and Dongjin based on the Student’s *t*-test at *p* < 0.01. **(D,E)** Phenotypic difference between Dongjin and T-DNA insertion lines.

To determine whether *OsD2* regulates LTG, we obtained two T-DNA insertion lines (PFG_3A-07238 and PFG_3A-07287) from the Kyung Hee University. PCR analysis confirmed the T-DNA insertions in the third intron (Figures 3A,B). Homozygous plants with T-DNA insertion in PFG_3A-07238 showed severe dwarfism with no seed setting, whereas heterozygous plants displayed a non-dwarf phenotype (Figure 3D, Supplementary Figure 1). The qRT-PCR results showed that *OsD2* expression in PFG_3A-07238 heterozygous plants was significantly lower than that in Dongjin plants (Figure 3C). On the other hand, PFG_3A-07287 heterozygous plants showed higher *OsD2* expression levels than Dongjin plants (Figure 3C). *OsD2* has two types of transcript form, *OsD2a* (LOC_Os01g10040.1) and *OsD2b* (LOC_Os01g10040.2). PFG_3A-07287 includes pGA2715 vector which harbors CaMV 35S enhancers which might have induced the overexpression of the *OsD2b* transcript, leading to higher expression of *OsD2b* in PFG_3A-07287. Therefore, we selected two heterozygous lines for *OsD2*-knockdown (*OsD2*-KD; line numbers: 9000-2 and 9011-14) and *OsD2*-overexpression (*OsD2*-OE; line numbers: 9002-6 and 9012-15). To check whether *OsD2*-KD and *OsD2*-OE make *OsD2a* or *OsD2b* transcript, PCR was conducted using cDNA of Dongjin, *OsD2*-KD, and *OsD2*-OE (Figure 3B). *OsD2a* transcript was observed in Dongjin while amplicon was not detected in *OsD2*-KD and *OsD2*-OE. *OsD2b* transcript was observed in all materials and *OsD2*-KD displayed lower expression of *OsD2* transcript than Dongjin. *OsD2*-OE showed higher expression of *OsD2b* transcript than Dongjin and *OsD2*-KD. The two *OsD2*-KD lines showed erect plant architecture with smaller grain size than Dongjin plants, whereas the *OsD2*-OE lines displayed an open plant architecture with longer grains than Dongjin plants (Figures 3D,E, Supplementary Figure 4).

**Figure 4:**
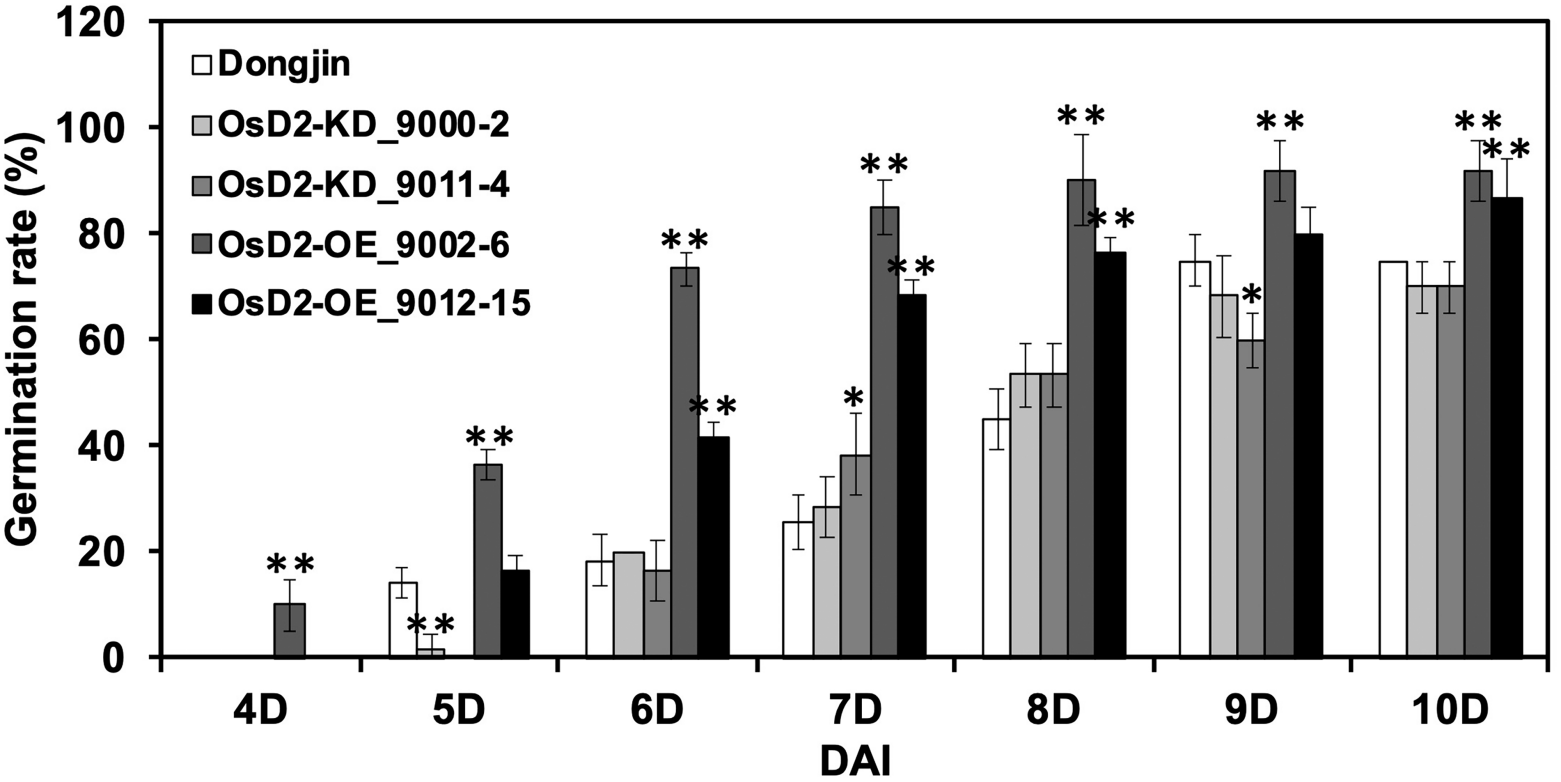
Low-temperature germinability of *OsD2* T-DNA insertion lines and Dongjin (DJ). Seeds of *OsD2*-KD (9000-2, 9011-4), *OsD2*-OE (9002-6, 9012-15), and Dongjin were incubated at 13°C for 10 days. Data are presented by mean ± SD (*n* = 3). * and ** indicate significant difference between each T-DNA insertion line and Dongjin at *p* < 0.05 and *p <* 0.01, respectively, based on the Student’s *t*-test.

The LTG test was performed using two T-DNA lines and wild-type Dongjin (Figure 4). At the optimal germination temperature, Dongjin, *OsD2*-KD, and *OsD2*-OE seeds showed 100% germination rates at three DAI (Supplementary Figure 5). The LTG of *OsD2*-OE was significantly higher than that of Dongjin and *OsD2*-KD. *OsD2*-OE (9002-6) started germination at four DAI, while the other lines started germination at 5 or 6 DAI, indicating that *OsD2* plays a role in LTG regulation. However, *OsD2*-KD lines displayed a lower germination rate than Dongjin at 5 DAI, while the LTG differences between *OsD2*-KD lines and Dongjin were not significantly different except at 7 and 9 DAI. This is possibly due to the genetic background effects of seeds obtained from the heterozygous plants.

### Haplotype analysis for *OsD2*

**Figure 5:**
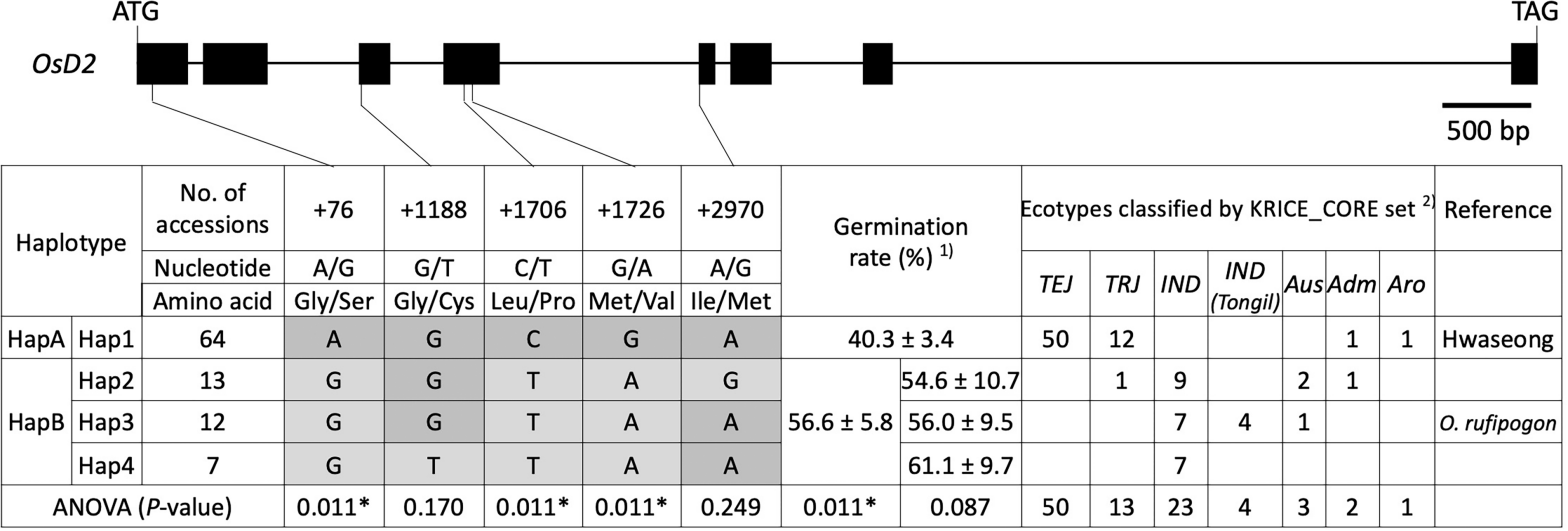
Haplotype analysis of *OsD2* using 96 rice accessions from the KRICE_CORE set. 1) Germination rate was presented as mean ± SE. 2) *TEJ*, temperate *japonica*; *TRJ*, tropical *japonica*; *IND*, *indica*; *IND* (Tongil), *indica* Tongil; *Aus*, Aus; *Adm*, admixture; and *Aro*, aromatic. * indicates significant difference at *p* < 0.05 based on one-way ANOVA.

To validate the allelic effects of *OsD2*, haplotype analysis was conducted using 96 rice accessions from the KRICE_CORE set (Supplementary Table 2). Four haplotypes (Hap1–4) were found when all five SNPs were considered (Figure 5). Hap1 contained 64 accessions, including 50 temperate *japonica*, 12 tropical *japonica*, one admixture, and one aromatic rice. Hwaseong was classified as Hap1, and this haplotype showed 40.3% LTG at 6 DAI. Hap2 had 13 accessions, including one tropical *japonica*, nine *indica*, two Aus, and one admixture. The rice accessions in Hap2 displayed an average of 54.6% LTG. *Oryza rufipogon* belonged to Hap3, along with seven *indica*, four *indica* (Tongil), and one Aus accession. Under low-temperature conditions, Hap3 had 56.0% mean germination rate. Seven *indica* accessions were classified as Hap4 and showed 61.1% mean germination rate at 13°C. ANOVA was performed to determine whether LTG was significantly different among the four haplotypes at *p* < 0.05 level. However, the difference was not significant, possibly because of a large amount of variation within the haplotypes (*p* = 0.087). We then evaluated the effects of each of the five SNPs to identify informative SNPs associated with the LTG variation. At three SNPs (+76_A/G, +1706_C/T, and +1726_C/T), the four haplotypes were classified into two groups (Hap1 and Hap2–4). At the second SNP (+1188_G/T), two haplotype groups, Hap1–3 and Hap4, were formed. Hap2 formed one group, and the other three haplotypes, Hap1, 3, and 4, formed the other group based on the fifth SNP (+2970_A/G). ANOVA showed that the difference in LTG between the two groups was significant (*p* = 0.011) at three SNPs (+76_A/G, +1706_C/T, and + 1726_C/T), but not significant at the second or fifth SNP (Figure 5). Therefore, Hap1 and Hap2–4 were formed as new haplotype group as HapA and HapB based on the three significant SNPs, respectively (Figure 5). These results indicate that three SNPs (+76_A/G, +1706_C/T, and + 1726_C/T) are informative in explaining the LTG variation, and these SNPs could be used in rice breeding programs for selecting parents with enhanced LTG.

### BR is associated with low-temperature germination

*OsD2*-KD and *OsD2*-OE plants showed several differences in plant architecture compared to Dongjin plants (Figures 3D,E). *OsD2*-KD plants flowered 1 week later than the control Dongjin plants, had erect leaves with a narrow leaf angle, and did not show dwarfism. In contrast, *OsD2*-OE plants were shorter in plant height than Dongjin plants and had bent leaves, indirectly implying a higher amount of BR.

**Figure 6:**
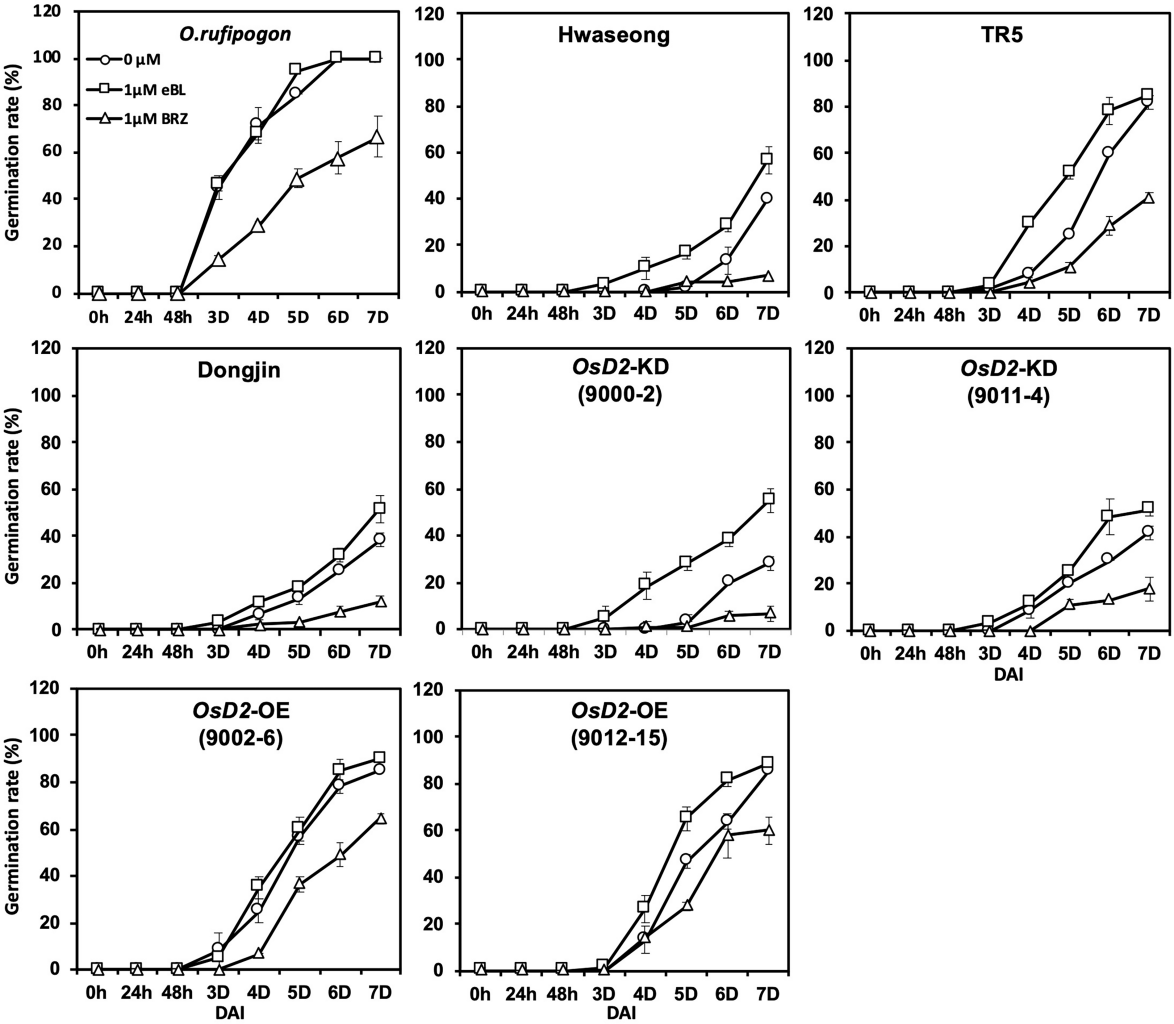
Comparison of the low-temperature germinability of eight plant materials under the exogenous brassinosteroid and brassinosteroid biosynthesis inhibitor treatment. Twenty-five seeds were incubated at 13°C with control (circle), 1 μM 24-epi-brassinolide (eBL; square), and 1 μM brassinazole (BRZ; triangle) for 7 days. Data are presented as mean ± SD (*n* = 3). DAI, days after incubation.

To determine whether BR content variation was responsible for the improved LTG in *OsD2*-OE, a feeding experiment was conducted. We investigated LTG following seed treatment with exogenous eBL and BRZ (a BL biosynthesis inhibitor; Figure 6). The low-temperature germination rates of Hwaseong, TR5, Dongjin, *OsD2*-KD (9000-2 and 9011-4), and *OsD2*-OE (9012-15) increased under eBL treatment, whereas the germination rates of *O. rufipogon* and *OsD2*-OE (9002-6) at low temperatures were not significantly different from the control condition. In contrast, BRZ treatment decreased the low-temperature germination rate of all lines. This experiment showed that the exogenous application of BR promoted LTG, whereas the application of a BR biosynthesis inhibitor decreased LTG. These results indicate that BR is associated with LTG.

### Expression patterns of BR biosynthesis and signaling genes

Endogenous BR concentration is a major factor regulating the expression of BR biosynthesis and signaling pathway genes. The plant architecture of *OsD2*-KD and *OsD2*-OE plants indirectly indicated the endogenous BR content. The expression patterns of genes involved in BR signaling and biosynthetic pathways were analyzed using transgenic plants (Supplementary Figure 6). The transcript levels of the receptor-like kinase *BRASSINOSTEROID INSENSITIVE 1* (*OsBRI1*), co-receptor kinase *BRI1-ASSOCIATED KINASE 1* (*OsBAK1*), *BRASSINAZOLE-RESISTANT 1* (*OsBZR1*), and *INCREASE LEAF INCLINATION 4* (*OsILI4*) were considerably lower in both *OsD2*-KD and *OsD2*-OE than in Dongjin (Supplementary Figure 6A). Lower expression of two *GLYCOGEN SYNTHASE KINASE* (*OsGSK2* and *OsGSK3*) and *DWARF* and *LOW TILLERING* (*OsDLT*) genes was observed in *OsD2*-KD lines than in Dongjin, whereas the expression of the these three genes was higher in *OsD2*-OE lines than in Dongjin (Supplementary Figure 6A). Among the major BR biosynthesis genes, higher expression levels of *OsD11, OsCPD1,* and *OsBR6ox* were detected in the *OsD2*-OE lines than in Dongjin, whereas the BR-deficient gene, *OsBRD1*, was downregulated in the *OsD2*-OE lines (Supplementary Figure 6B). Conversely, the *OsD2*-KD lines showed lower expression of *OsD11* and *OsBRD1* than Dongjin. The different expression levels of BR signaling and biosynthesis genes in T-DNA insertion lines might be associated with the difference in endogenous BR content, leading to the variation in LTG, plant architecture, and seed size in the T-DNA lines.

## Discussion

Low-temperature germinability is an important trait for direct-seeded rice cultivation. Because of the significance and complex nature of LTG in rice, many genetic approaches have been used to identify genes involved in LTG (Satoh et al., 2016; Wang et al., 2018; Yang et al., 2020). To date, two genes, *qLTG3-1* and *OsSAP16* have been characterized using biparental and GWAS approaches, respectively (Fujino et al., 2008; Wang et al., 2018). In this study, we demonstrated that the BR biosynthesis gene *OsD2* is the causal gene for the LTG QTL, *qLTG1*. Two introgression lines, TR5 and TR20 (BC_3_F_6_), harboring the *O. rufipogon qLTG1* allele, showed significantly enhanced LTG compared to Hwaseong (Shim et al., 2020). Map-based cloning enabled us to delimit the QTL to a ~30-kb region flanked by two markers, CRM23 and CRM22. The region has four genes: prenylated rab acceptor (LOC_Os01g10010), ectonucleotide pyrophosphatase/phosphodiesterase family member 1 (LOC_Os01g10020), hypothetical protein (LOC_Os01g10030), and cytochrome P450 (LOC_Os01g10040, *OsD2*). To identify the causal gene for *qLTG1*, gene sequencing, expression analysis, and transgenic approaches were employed. Gene expression analysis indicated that the *O. rufipogon* and TR5 seeds showed higher *OsD2* expression than the Hwaseong seeds during germination, whereas the three lines (Hwaseong, *O. rufipogon*, and TR5) did not show any consistent expression pattern in the other three genes (LOC_Os01g10010–LOC_Os01g10030). Based on the gene expression analysis, *OsD2* was selected as the candidate gene for *qLTG1*. Sequence comparison of *OsD2* between Hwaseong and *O. rufipogon* revealed five SNPs in the exon region and 14 sequence variants in the promoter region. To confirm the association of *OsD2* with LTG, we employed two T-DNA insertion lines: *OsD2*-OE and *OsD2*-KD plants in the Dongjin background. The LTG test using the two T-DNA lines and Dongjin showed that *OsD2*-OE had significantly higher LTG than Dongjin and *OsD2*-KD. To determine whether BR is responsible for the improved LTG in *OsD2*-OE, feeding experiments were conducted using exogenous eBL and BRZ (Figure 6). The low-temperature germination rates of Hwaseong, TR5, Dongjin, *OsD2*-KD (9000-2 and 9011-4), and *OsD2*-OE (9012-15) increased under eBL treatment, whereas the germination rates of *O. rufipogon* and *OsD2*-OE (9002-6) at low temperatures were not significantly different from the control condition. In contrast, BRZ treatment resulted in a decrease in the low-temperature germination rate of all lines. Studies have reported that LOC_Os01g10040 (*OsD2*) plays an important role in regulating plant height, grain size, and leaf angle (Hong et al., 2003; Li et al., 2013a; Fang et al., 2016). Our findings indicated that *OsD2* is also associated with LTG variation in rice.

The *ebisu dwarf* (*dwarf2* or *d2*), an *OsD2* gene mutant, has been characterized, and several *d2* alleles have been reported (Hong et al., 2003; Li et al., 2013a; Fang et al., 2016). Among these alleles, *d2-3*, *d2-4*, *d2-6*, and *ccdd1* showed severe dwarfism with undesirable traits, making their utilization impractical in breeding programs. Although the *d2-1*, *d2-2,* and *smg11* alleles of *OsD2* displayed non-dwarf phenotypes, plants with these alleles had reduced plant height, small grains, and erect plant architecture (Hong et al., 2003; Fang et al., 2016). To date, many studies have reported the function of BRs as a class of plant steroidal hormones and have focused on the effect of BR on agronomic and morphological traits (Fujioka and Yokota, 2003; Wu et al., 2008). In contrast, we analyzed the association of *OsD2* with germinability under stress conditions and found that the *O. rufipogon OsD2* allele improved LTG in the cultivated rice background without any deleterious phenotypes such as dwarfism. In addition, two introgression lines TR5 and TR20 harboring the *O. rufipogon qLTG1* allele showed improved agronomic traits, such as increased grain size, spikelets per panicle, 1,000-grain weight, and grain yield whereas their tall statue are unfavorable because of lodging (Yun et al., 2016, Supplementary Figures 7, 8). Although it is unclear whether these changes in agronomic traits are due to the pleiotropic effect of *OsD2* or other tightly linked genes, these results support the fact that wild rice is a valuable resource for improving yield stability and widening genetic diversity in rice breeding programs (Xiao et al., 1996; Yeo et al., 2014; Yun et al., 2016; Shim et al., 2019).

Haplotype analysis of 96 rice accessions with five non-synonymous SNPs in *OsD2* was performed to determine the association between haplotypes and LTG (Figure 5). When the five SNPs were combined, the 96 accessions were classified into four haplotypes (Hap1–4). However, the difference in LTG levels among the four haplotypes was not significant. Then, each of the five SNPs was considered individually, and the 96 accessions were classified into two haplotypes. At three SNP sites (+76, +1,706, and +1,726), Hap1 and Hap2–4 formed two different haplotypes (HapA and HapB), and two groups showed significant differences in LTG (*p* < 0.05, ANOVA). Interestingly, rice accessions in HapB shared the same genotype at +76, +1,706, and +1,726 SNPs, and three SNPs each showed significant differences in LTG based on ANOVA (*p* = 0.011; Supplementary Figure 3). It remains to be determined whether the amino acid sequence of *OsD2* between the two lines is directly associated with LTG. However, the possibility that the sequence difference in the *OsD2* promoter region is responsible for the variation in LTG cannot be ruled out. Although further studies on protein structure and enzymatic experiments are needed to answer this question, the molecular markers for +76, +1,706, and +1,726 SNPs would be useful in selecting high-LTG germplasms together with the previously designed *qLTG3-1* functional markers (Shim et al., 2020). In addition, pyramiding two *O. rufipogon* alleles at *qLTG1* and *qLTG3* will be an effective strategy for developing direct-seeding varieties with high LTG.

Two T-DNA insertion lines were used for *OsD2* characterization (Jeon et al., 2000; Jeong et al., 2006). Generally, homozygous T-DNA insertion lines are used to examine the effects of gene knockout or overexpression. However, in our study, we employed heterozygous T-DNA plants for phenotypic evaluation because the homozygous T-DNA insertion plants showed severe dwarfism and failed to set seeds. The absence of homozygous seeds made us to employ segregating seeds in the germination test and this could have affected the accuracy of the germination test. The seeds harvested from heterozygous plant will be segregating with 1:2:1, 3:1, or 1:3 ratio depending on the gene action (additive, dominant, or recessive). Therefore, 75% of seeds of heterozygous plants have at least one-copy T-DNA insertion and the number of seeds showing higher or lower *OsD2* expression than the WT Dongjin would be different depending on the gene action. This might have affected the accuracy of the LTG test especially in *OsD2*-KD lines.

The heterozygous plants (*OsD2*-KD) showed lower *OsD2* expression similar with other BR-deficient mutants phenotypes, such as *d2-1* and *d2-2,* and displayed erect leaves with decreased plant height and grain size (Figure 3; Hong et al., 2003). The *OsD2*-OE plants showed a higher expression of *OsD2,* although the insertion site of T-DNA was located near the site for *OsD2*-KD. It is possible that the CaMV 35S promoter enhancer in the vector border may have activated the expression of *OsD2b* transcript form (Jeong et al., 2006; Li et al., 2013a). Similar studies using T-DNA insertion lines for overexpression and decreased gene expression have been reported. Two T-DNA insertion mutants, *oswrky5-D* and *oswrky5* showed increased and decreased expression levels of *OsWRKY5*, respectively, and the expression of *OsWRKY5* was positively associated with the leaf senescence phenotype (Kim et al., 2019).

Studies have reported that BRs play an important role in promoting seed germination (Leubner-Metzger, 2001; Steber and McCourt, 2001). Leubner-Metzger (2001) observed that brassinolide promotes tobacco seed germination under both light and dark incubation conditions. BR signaling is required to overcome the inhibition of germination by ABA, suggesting that BR stimulates seed germination (Steber and McCourt, 2001). It has been demonstrated that the exogenous application of BRs enhanced the seed germination rate under stress conditions in rice and cucumber (Anuradha and Rao, 2001; Wang et al., 2011). However, the effects of BR under low-temperature stress conditions have not yet been examined. In our feeding experiment, the application of 1 μM eBL improved the germination rate of Hwaseong, TR5, Dongjin, *OsD2*-KD (9000-2 and 9011-4), and *OsD2*-OE (9012-15) at low temperatures (Figure 6). In contrast, no significant difference was observed in in the germination rates of *O. rufipogon* and *OsD2*-OE (9002-6). This was possibly due to the high level of endogenous BR in the two lines, and the eBL treatment did not contribute to the increase in LTG compared to the control. On the other hand, BRZ treatment decreased LTG in all tested lines. These results clearly indicate that BR is associated with LTG.

In this study, the expression patterns of some BR signaling and biosynthesis genes were not consistent with those reported in previous studies. For example, the lower expression of *OsILI4* (*BRASSINOSTEROID UPREGULATED1, OsBU1*), encoding Helix–loop–Helix protein involved in BR signaling, in both *OsD2*-KD and *OsD2*-OE is not consistent with previous reports. Sakamoto et al. (2012) reported that the relative *OsBU1* expression levels in *d2-1*, *d2-3*, *d2-4,* and *d2-6* mutants were not significantly different from their corresponding wild types. Tanaka et al. (2009) reported that *OsBU1* overexpression plants showed enhanced bending of the lamina joint and enlarged grain size, which was not consistent with the characteristics of *OsD2*-OE lines in the present study. However, Li et al. (2013a) observed a decreased expression of *OsBU1* in the *OsD2* null mutant (*csdd1*), which is consistent with our observations. In our study, BR synthesis genes *OsD11* and *OsBRD1* which are regulated by the BR signaling gene *RELATED TO ABI3/VP1-LIKE1* (*RAVL1*), showed significantly decreased expression in *OsD2*-KD lines (Supplementary Figure 6; Je et al., 2010). However, *OsD2*-OE lines displayed increased expression levels of *OsD11* and decreased expression levels of *OsBRD1*. Li et al. (2013a) reported that the *OsD2* null mutant *csdd1* showed increased expression levels of *OsD11* and *OsDWARF* (*OsBRD1*) compared to wild-type plants. This discrepancy in the expression pattern of genes involved in BR signaling and biosynthesis may be due to the use of heterozygous *OsD2*-KD and *OsD2*-OE plants.

Low-temperature stress is a challenge in crop cultivation. In this study, a new QTL, *qLTG1*, controlling LTG was detected in an introgression line (TR5) derived from a cross between the *japonica* variety “Hwaseong” and the wild rice (*O. rufipogon*). Using map-based cloning, transgenic approaches, and gene expression analysis, we demonstrated that the rice BR biosynthesis gene *OsD2* is the causal gene for *qLTG1*. The *O. rufipogon OsD2* allele improved LTG in the cultivar background without any deleterious phenotypes. These results will benefit rice breeding programs by assisting in developing strategies to maximize the exploitation of invaluable genes from interspecific or intraspecific crosses.

## Data availability statement

The original contributions presented in the study are included in the article/Supplementary material, further inquiries can be directed to the corresponding author.

## Author contributions

SK, K-CS, and S-NA designed the experiments and wrote the manuscript. SK and K-CS carried out phenotype analysis (LTG test). H-SL and Y-AJ performed qRT-PCR analysis. CA and NL conducted the agronomic traits investigation. All authors contributed to the article and approved the submitted version.

## Funding

This work was carried out with the support of “Cooperative Research Program for Agriculture Science and Technology Development (Project No. PJ015757)” Rural Development Administration, Republic of Korea.

## Conflict of interest

The authors declare that the research was conducted in the absence of any commercial or financial relationships that could be construed as a potential conflict of interest.

## Publisher’s note

All claims expressed in this article are solely those of the authors and do not necessarily represent those of their affiliated organizations, or those of the publisher, the editors and the reviewers. Any product that may be evaluated in this article, or claim that may be made by its manufacturer, is not guaranteed or endorsed by the publisher.

## Footnotes

^1^ http://rice.uga.edu
