# Supplementary material for: Brassinosteroid biosynthesis gene OsD2 is associated with low-temperature germinability in rice
Source: Front Plant Sci. 2022 Sep 20;13:985559. doi: 10.3389/fpls.2022.985559 (PMC9530605; doi:10.3389/fpls.2022.985559)
Supplement: Supplementary file 1 [file Data_Sheet_1.docx]

**Supplementary Figure 1**. Phenotype of T-DNA insertion homozygous genotype plants and the wild type (Dongjin).

**Supplementary Figure 2**. Gene expression analysis of four candidate genes for *qLTG1*. Four samples each pf Hwaseong, *O. rufipogon*, and TR5 seeds, taken during early seed germination stages (0, 24, 48, and 72 h after incubation) were used. qRT-PCR was conducted to determine the relative transcript level of genes and *OsTMP* was used for normalization. Error bars indicate standard deviation.

**Supplementary Figure 3**. (**A**) Sequence comparison of the *OsD2* exon region between Hwaseong and *O. rufipogon*. Black boxes indicate exons and white boxes indicate untranslated region. Amino acid codes written in red colors denote the different amino acids between Hwaseong and *O. rufipogon* caused by non-synonymous mutation. (**B**) Schematic representation of two forms of OsD2 protein (D2a and D2b) modified from Li et al. (2013).

**Supplementary Figure 4**. Comparison of grain size of two T-DNA insertion lines (*OsD2*-KD and *OsD2*-OE) together with Dongjin (DJ), Hwaseong (HS) and TR5. (**A**) Comparison of seed size, (**B**) grain width, and (**C**) grain length of plant materials. * and ** indicate significant difference between Hwaseong and TR5, and each T-DNA insertion line and Dongjin based on the Student’s *t*-test at *p* < 0.05 and *p* < 0.01, respectively.

**Supplementary Figure 5**. Germination rate of T-DNA insertion lines, *OsD2*-KD (CR9000-2 and 9011-4) and *OsD2*-OE (CR9002-6 and CR9012-15) with Dongjin (DJ) at optimal temperature (28 ℃). DAI: days after incubation.

**Supplementary Figure 6**. (**A**) Expression levels of BR-signaling pathway genes in Dongjin and *OsD2* mutants. (**B**) Expression levels of BR-biosynthesis genes. Young seedings were used for RNA extraction. *OsTMP* was used for gene normalization. Data are presented as mean ± standard deviation. * and ** indicate significant difference between each T-DNA insertion line and Dongjin based on the student’s *t*-test at *p* < 0.05 and *p* < 0.01, respectively.

**Supplementary Figure 7**. Comparison of (**A**) grain shape and (**B**) panicles among Hwaseong, *O. rufipogon*, and TR5.

**Supplementary Figure 8**. Evaluation of agronomic traits in Hwaseong and two introgression lines which harbor *qLTG1*. Comparison was made between Hwaseong and each introgression line. * , ** and *** indicate significant difference between each line and Hwaseong based on the Student’s *t*-test at *p* < 0.05, *p* < 0.01 and *p* < 0.001, respectively. NS: non-significant.
